# Community resilience and stroke outcomes in older adults: beyond rural-urban classifications

**DOI:** 10.3389/fpubh.2025.1700006

**Published:** 2025-12-12

**Authors:** Ruaa Al Juboori, Emily Welker, Marie Barnard, Keith Anderson

**Affiliations:** 1 Public Health, School of Applied Sciences, The University of Mississippi, Oxford, MS, United States; 2 Pharmacy Administration, School of Pharmacy, The University of Mississippi, Oxford, MS, United States; 3 Social Work, School of Applied Sciences, The University of Mississippi, Oxford, MS, United States

**Keywords:** stroke burden, resilience domains, Medicare beneficiaries, geospatial analysis, rural-urban trajectories, BRIC index, health disparities

## Abstract

**Introduction:**

Stroke is a leading cause of death and disability among older U. S. adults, with persistent geographic disparities. The role of community resilience, and whether its effects differ across changing rural-urban county trajectories, remains underexamined. This study assessed associations between county-level resilience domains and stroke burden among Medicare fee-for-service beneficiaries aged ≥65 and evaluated variation across stable rural, stable urban, rural-to-urban transitioning, and urban-to-rural deurbanizing counties.

**Methods:**

This ecological study analyzed 3,100 U. S. mainland counties. County trajectories were derived using 2010 and 2020 Rural-Urban Continuum Codes. Resilience was measured using the Baseline Resilience Indicators for Communities (BRIC) index, which includes social, economic, community capital, institutional, infrastructure, and environmental domains. Correlation and multivariable regression models examined associations between resilience domains and stroke burden (2022). Spatial clustering was assessed using Local Indicators of Spatial Association (LISA), which identifies high-high and low-low groupings where counties have values similar to their neighbors.

**Results:**

Mean stroke burden (% of Medicare FFS beneficiaries). was 5.5% (SD = 1.47) and increased by 2.17 percentage points (SD = 1.90) from 2014 to 2022, with the highest levels in urban and deurbanizing counties. Social resilience was consistently associated with lower stroke burden across all trajectories. Infrastructure and environmental resilience were also inversely associated, though effects varied by trajectory. Economic and institutional resilience were positively associated with burden in the overall and rural models. In multivariable analyses, social resilience remained the strongest negative predictor across all strata and the only consistently significant domain in transitioning and deurbanizing counties. Spatial analysis showed high-high stroke clusters in the Southeast (Stroke Belt), while most resilience domains exhibited low-low clusters in the same regions.

**Conclusion:**

Social and infrastructural resilience were the most consistent protective factors for stroke burden among older adults, whereas positive associations for economic and institutional domains likely reflect contextual or measurement characteristics. Overlapping low-resilience and high-stroke clusters in the Stroke Belt highlight the need for trajectory-based, resilience-informed strategies to reduce geographic disparities among older Medicare beneficiaries.

## Introduction

Stroke is a leading chronic condition among older adults and a major cause of death and disability in the United States. Approximately three-quarters of all strokes occur in individuals aged 65 years and older, with ischemic strokes accounting for the majority (1). The risk of stroke increases sharply with age, doubling every decade after age 55. In 2020, the age-adjusted death rate for stroke among adults aged 65 and over was 260.5 per 100,000 (2). This burden is compounded by high rates of cardiovascular disease in the same age group, which shares risk factors and pathophysiologic pathways with stroke.

National data also indicate that stroke mortality and hospitalization rates remain highest in the South, particularly in the Stroke Belt, and affect different racial/ethnic groups (3). As the U. S. population ages, the number of individuals at risk will continue to rise. The population aged 65 years and older is projected to more than double from 40.2 million in 2010 to 88.5 million by 2050 (U. S. Department of Commerce).

This demographic shift is expected to increase the health and economic burden of stroke, driving demand for prevention, acute care, and long-term rehabilitation services. Rural-urban disparities in stroke burden remain a major public health challenge in the United States, especially among older adults. Multiple studies have found that stroke mortality and hospitalization rates are consistently higher in rural counties compared to urban ones (4, 5). This gap is partly explained by the greater prevalence of key vascular risk factors in rural communities, including hypertension, diabetes, and heart disease (6). Limited access to care also plays an important role, rural areas often face shortages of primary care providers and specialists, longer travel times to hospitals and fewer facilities with advanced stroke treatment capabilities (7, 8). These structural barriers can delay diagnosis and treatment, leading to worse outcomes. Geographic disparities are most evident in the southeastern “Stroke Belt,” where many rural counties report some of the nation’s highest rates of stroke-related death and disability (3, 9). Importantly, patterns of risk and outcomes may differ in transitioning counties, those shifting from rural to urban or from urban to rural, due to changes in population demographics, economic conditions, and healthcare infrastructure (10, 11). Understanding how these factors interact is essential for identifying where prevention and resource allocation efforts can have the greatest impact.

While rural-urban disparities highlight where stroke burdens are concentrated, resilience factors can help explain why some counties perform better than others despite high levels of risk. Community resilience, the capacity to adapt and maintain function in the face of adversity, operates at both the individual and community level. Among older adults in the U. S., individual level resilience factors such as strong social networks, access to supportive relationships and effective coping strategies have been linked to better recovery and quality of life after major health events (12–14). At the same time, community-level conditions such as economic stability, safe housing, accessible healthcare, and environmental safety can either buffer against or exacerbate poor stroke outcomes. Therefore, the objective of this study assesses associations between community resilience factors measured at the county-level and stroke burden among Medicare fee-for-service beneficiaries aged ≥65, and to assess how these associations vary across stable rural, stable urban, rural to urban transitioning, and urban to rural deurbanizing counties.

## Methods

### Study design and variables

This study employed an ecological, county level observational design to explore how community resilience factors are associated with stroke burden (% of Medicare FFS beneficiaries) among adults aged ≥65 across rural, urban, and transitioning counties in the contiguous United States. We analyzed 3,100 U. S. mainland counties.

### Variables

#### Stroke

The stroke variable in this study represents the percentage of Medicare fee-for-service beneficiaries who had a documented stroke diagnosis in CMS administrative claims for that calendar year. Stroke burden was defined as the percentage of Medicare fee-for-service (FFS) beneficiaries aged ≥65 years diagnosed with stroke in a given county. This measure is obtained from the Centers for Medicare & Medicaid Services (CMS) Chronic Conditions Data Warehouse (CCW), made available through PolicyMap. CMS classifies a beneficiary as having stroke when a Medicare FFS enrollee has a claim indicating receipt of services or treatment for stroke. This metric reflects the share of FFS beneficiaries with a documented stroke diagnosis and is not equivalent to epidemiologic prevalence in the general population. The change in stroke burden variable represents the difference in percentage points between 2022 and 2014. The stroke variable was obtained from PolicyMap, which compiles county-level data on Medicare fee-for-service beneficiaries from the Centers for Medicare & Medicaid Services (CMS) Chronic Conditions Data Warehouse/Geographic Variation Public Use Files (15). It is important to note that Medicare introduced modifications to its enrollment and reporting system in 2013 (16). To examine temporal change, we calculated the difference between the 2022 and 2014 CMS stroke-diagnosis percentages. The year 2014 was chosen as the baseline because CMS updated its reporting and classification systems in 2013, affecting comparability with earlier years. Stroke burden was measured using Medicare fee-for-service (FFS) claims, which reflect diagnosed stroke events recorded in administrative billing data.

#### Rural Urban Continuum Codes classification

**Figure 1:**
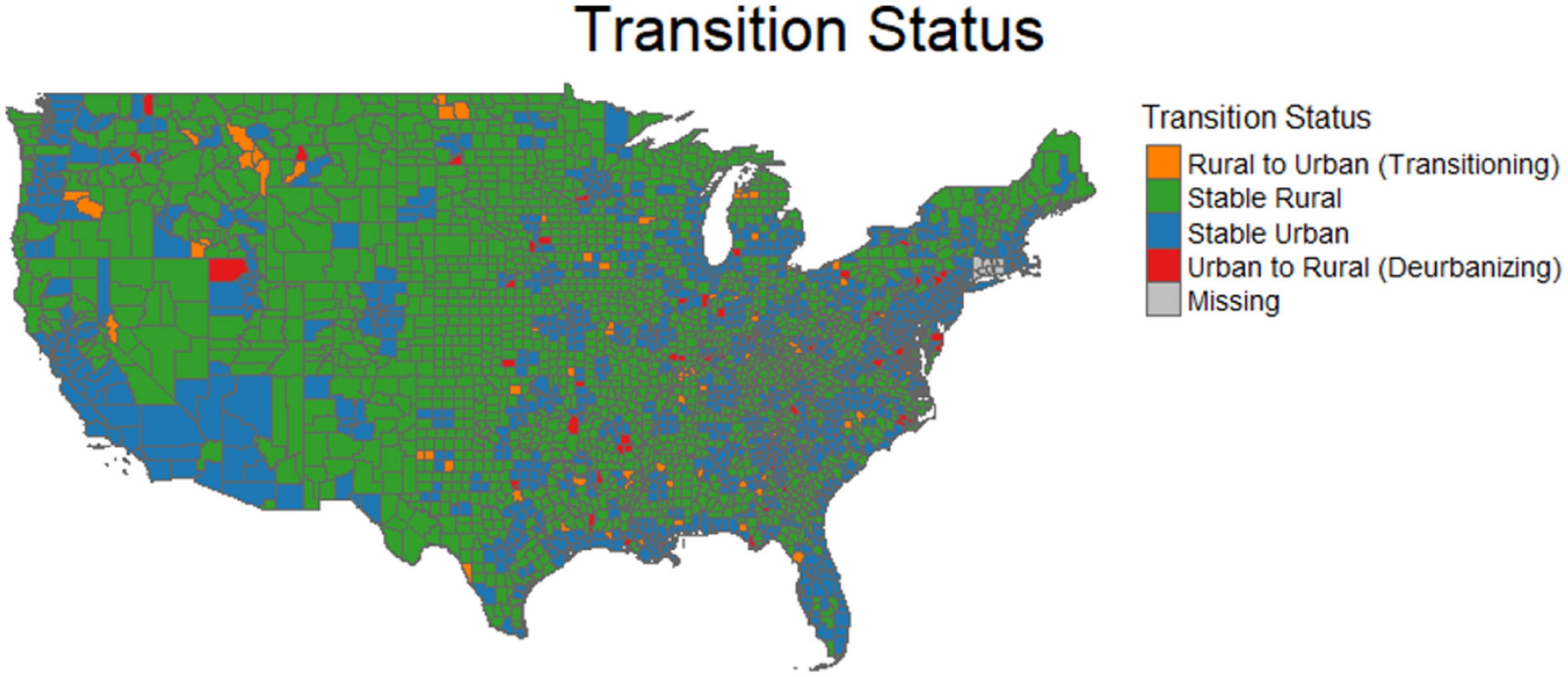
Distribution of U. S. counties by rural-urban classification (2010–2020).

Counties were classified as stable rural, stable urban, rural-to-urban transitioning, or urban-to-rural deurbanizing using the 2020 and the 2010 Rural Urban Continuum Codes (RUCC) codes. Rural-urban classifications were based on the USDA Economic Research Service Rural-Urban Continuum Codes (2013, 2023) (17, 18). To assess county level rural-urban dynamics over the past decade, we used the 2010 and 2020 RUCC. Counties were first recategorized into two groups based on the original RUCC 1–9 scale: urban (codes 1–3) and rural (codes 4–8). Only U. S. mainland counties were included in the analysis. It is important to note that some exceptions were addressed due to the mismatch between 2010 and the 2020 RUCC codes: Nine coding areas (09110–09190) representing Connecticut planning regions were excluded because they did not align with county based RUCC coding. Oglala Lakota County, SD (formerly Shannon County) was retained after verifying consistent identifiers across datasets. Counties missing RUCC data were also excluded. After these adjustments, the final analytic sample comprised 3,100 U. S. mainland counties. Comparing RUCC classifications between 2010 and 2020, most counties remained stable in their status: (1875: 60.48%) were consistently rural and (1,101: 35.52%) consistently urban. A smaller proportion of counties experienced change, with (72: 2.32%) transitioning from rural to urban and (52: 1.68%) shifting from urban to rural (deurbanizing). Please refer to Figure 1 for a visual representation of the counties’ classification distribution.

#### Baseline Resilience Indicators for Communities

Resilience was measured using BRIC framework that provides six domain-specific measures of resilience: social, economic, community capital, institutional, infrastructure, and environmental. These domains are constructed from county-level indicators compiled by the University of South Carolina’s Hazards Vulnerability & Resilience Institute. Each indicator is normalized to a common 0–1 scale before being averaged to produce a domain score, and the six domains are then summed to create an overall resilience index. Higher scores reflect stronger resilience, while lower scores indicate relative vulnerability (19). The BRIC domains demonstrated expected spatial patterns and internal consistency, consistent with prior studies that have applied and validated BRIC as a reliable county-level resilience measure in population and hazard-related research (20, 21).

Social resilience reflects the extent to which a population has the social and health resources necessary to withstand shocks. It includes factors such as educational attainment, health insurance coverage, access to healthcare providers, transportation and communication capacity, and language/communication characteristics (including limited English proficiency). Counties with lower values in this domain generally have weaker access to health and social services, while those with higher scores tend to have more robust support systems.

Economic resilience represents the stability and diversity of a county’s economic base. Indicators capture labor force characteristics, income conditions, the mix and size of local businesses, and reliance on potentially vulnerable economic sectors. Low scores suggest fragile or undiversified economies; high scores reflect stronger, more adaptable economic structures. Community capital encompasses the strength of social networks and civic involvement. This domain considers participation in civic organizations, volunteer activity, religious or community involvement, and other forms of social cohesion. Low scoring counties may have limited civic engagement, whereas high-scoring counties tend to show more collective involvement and stronger local networks. Institutional resilience describes the ability of local governments and institutions to plan for, manage, and respond to disruptions. It includes factors such as emergency management capacity, hazard mitigation planning, administrative coordination, and the availability of institutional support. Lower values point to limited institutional capacity; higher values reflect stronger governance and planning systems (19).

Infrastructure resilience relates to the condition and availability of essential systems, including transportation networks, healthcare facilities, broadband and communication infrastructure, and shelter or housing conditions. Counties with lower infrastructure resilience may face limited mobility, reduced healthcare access, or weaker digital connectivity, while higher scores indicate more developed and reliable built systems. Environmental resilience captures ecological and land-use characteristics, including natural resource availability, environmental quality, land-use patterns, and ecological buffers. Lower scores indicate environmental stress or limited natural protection, whereas higher scores reflect more stable ecological conditions. (19). Demographic covariates such as age structure, race/ethnicity inequality, gender income inequality, education, disability, English proficiency, and healthcare access were not added as separate covariates because these constructs are already embedded within BRIC’s composite domains. The Social domain incorporates educational attainment, health insurance coverage, disability status, transportation/communication access, food security, physician availability, and pre-retirement age structure (15–65 population). The Economic domain includes measures of racial/ethnic income equality, gender income equality, employment, and economic diversity. Because BRIC domains mathematically integrate these sociodemographic and structural characteristics, entering them independently would duplicate underlying indicators, introduce multicollinearity, and obscure the interpretation of resilience domains. We therefore followed BRIC’s conceptual design, treating its domains as composite measures that already reflect demographic and structural context. Stroke burden was obtained from PolicyMap’s processed CMS layer titled “Percent of Medicare Fee-for-Service Beneficiaries Diagnosed with Stroke,” available for both 2014 and 2022. BRIC scores and all six BRIC domain indicators were extracted from (19) “Baseline Resilience Indicators for Communities (BRIC 2020)” layers. All PolicyMap layers were accessed and downloaded in August 2025 (15).

Please refer to Table 1 for a more detailed description of the study variables and the data sources.

**Table 1:** Study variables.

Variables	Variable description	Measurement type	Role	Data source
Outcomes				
Stroke burden between 2014 and 2022	Percent of Medicare fee-for-service beneficiaries who are diagnosed with stroke, as of 2022.	Continuous	Outcome	(15)
Stroke 2022	Difference between Percent of Medicare fee-for-service beneficiaries who are diagnosed with stroke, 2022 vs. 2014.	Continuous	Outcome	(15)
RUCC				
RUCC 2010	2010 Rural-Urban Continuum Codes	Categorical	Stratifier	(17)
RUCC 2020	2020 Rural-Urban Continuum Codes	Categorical	Stratifier	(18)
Predictors: resilience metrics				
BRIC	2020 score of overall resilience to natural hazards as of 2020.	Continuous	Predictor	(15, 19)
Social	2020 score of social resilience to natural hazards as of 2020.	Continuous	Predictor	(15, 19)
Economic	2020 score of economic resilience to natural hazards as of 2020.	Continuous	Predictor	(15, 19)
Infrastructural	2020 score of infrastructural resilience to natural hazards as of 2020.	Continuous	Predictor	(15, 19)
Community capital	2020 score of community capital resilience to natural hazards as of 2020.	Continuous	Predictor	(15, 19)
Institutional	2020 score of institutional resilience to natural hazards as of 2020.	Continuous	Predictor	(15, 19)
Environmental	2020 score of environmental resilience to natural hazards as of 2020	Continuous	Predictor	(15, 19)

### Statistical analyses

We conducted descriptive analyses to examine the mean and standard deviation of the outcome variables and the study predictors for all counties and per the four levels of the rural, urban, transitioning rural to urban and deurbanizing from urban to rural countries. Group differences in stroke burden, change in stroke burden, and resilience domains across the four county trajectory categories (urban, rural, transitioning, and deurbanizing) were tested using one-way ANOVA. Then we conducted correlation analysis to examine the association between the resilience metrics and the study outcomes (stroke 2022 and Stroke difference between 2022 and 2014). We fit multivariable linear regression analyses models that examine associations between resilience domains and the outcomes including only the variables that showed significant association in the bivariate correlation analyses. Before model fitting, we assessed the distribution of both outcome variables (stroke burden in 2022 and the change in stroke burden from 2014 to 2022). Both outcomes were approximately normally distributed with no severe skewing.

For spatial analysis, we first created color-shaded maps to visualize the geographical distribution of the study variables. To further assess spatial clustering and identify statistically significant hot and cold spots, we created Local Indicators of Spatial Association (LISA) maps. These maps show areas of high-high, low-low, high-low, and low-high associations. All analyses were conducted in R software (version 4.5.1), using a queen contiguity weight matrix, with the level of statistical significance set at *p* < 0.05. A replication dataset containing all counties with FIPS codes, RUCC based trajectory assignments is provided in Supplementary File 1. In addition to LISA, we calculated global Moran’s I for stroke burden (% of Medicare FFS beneficiaries), change in stroke burden, and each BRIC resilience domain to assess overall spatial autocorrelation across all U. S. counties. Global Moran’s I statistics were computed using a first-order queen contiguity weight matrix, which defines neighbors as counties sharing either a border or a vertex. Higher positive Moran’s I values reflect stronger spatial clustering, while values near zero indicate random spatial patterns. For LISA, each county is categorized into one of four types of spatial clusters. High-high clusters indicate counties with high values of a variable that are adjacent to neighboring counties that also have high values, which represents spatial hot spots. Low-low clusters represent counties with low values surrounded by neighbors with similarly low values, which form spatial cold spots. High-low clusters occur when a county with a high value is surrounded by counties with low values, which suggest a spatial outlier. Low-high clusters indicate the opposite, a county with a low value surrounded by high-valued neighbors, also represents a spatial outlier. These classifications help identify areas where geographic patterns of stroke burden and resilience are spatially concentrated or deviate from their surroundings. Although stroke and resilience indicators were drawn from different years, integrating temporally proximate datasets is a widely accepted approach in geospatial epidemiology, where county-level characteristics change slowly over time (22, 23).

## Results

Tables 2, 3 show the descriptive analyses of the variable and the results of the correlation analysis of the study variables and the stroke burden (% of Medicare FFS beneficiaries). For stroke, across all counties (*n* = 3,100), mean stroke percentage was 5.50% (SD = 1.47%). One-way ANOVA tests were used to compare mean differences in stroke burden and resilience metrics across the four county trajectory groups, and the resulting *p*-values are reported in Table 2. Negative correlations were observed for social (*r* = −0.24, *p* < 0.001) and infrastructural resilience (*r* = −0.175, p < 0.001). In rural areas (*n* = 1,875), mean stroke burden (% of Medicare FFS beneficiaries) was 5.27 (SD = 1.54). Negative correlations were observed for social (*r* = −0.35, *p* < 0.001) and infrastructural resilience (*r* = −0.35, *p* < 0.001).

**Table 2:** Descriptive statistics of the study variables, *N* = 3,100.

Variables	Overall		Urban		Rural		Transitioning (rural to urban)		Deurbanizing (urban to rural)		*p*-value
	3,100		1,101		1875		72		52		
	100%		35.52%		60.48%		2.32%		1.68%		
	Mean	SD	Mean	SD	Mean	SD	Mean	SD	Mean	SD	
Stroke burden (% of Medicare FFS beneficiaries)	5.50	1.47	5.87	1.24	5.27	1.54	5.69	1.46	5.79	1.4	<0.01
Change in stroke burden (percentage point difference, 2022–2014)	2.17	1.9	2.24	0.96	2.13	1.32	2.25	1.02	2.06	1.14	0.086
Resilience metrics											
BRIC	2.59	0.14	2.65	0.12	2.56	0.13	2.58	0.16	2.6	0.14	<0.01
Community Capital	0.35	0.05	0.34	0.05	0.36	0.05	0.35	0.05	0.35	0.04	<0.01
Economic	0.47	0.04	0.5	0.03	0.46	0.04	0.47	0.03	0.47	0.04	<0.01
Environment	0.53	0.04	0.52	0.05	0.53	0.04	0.54	0.05	0.54	0.05	<0.01
Infrastructure	0.25	0.05	0.27	0.05	0.24	0.05	0.23	0.05	0.24	0.05	<0.01
Institution	0.37	0.03	0.39	0.02	0.37	0.03	0.38	0.03	0.39	0.02	<0.01
Social	0.61	0.05	0.64	0.04	0.6	0.04	0.61	0.05	0.61	0.04	<0.01

*p*-values reflect one-way ANOVA tests comparing mean differences across county trajectory groups.

**Table 3:** Correlation results of study variables with prevalence of stroke in 2022.

	Overall (*n* = 3,100)		Urban (*n* = 1,101)		Rural (*n* = 1,875)		Transitioning (rural to urban) (*n* = 72)		Urban to rural (deurbanizing) (*n* = 52)	
	Corr.	*P*	Corr.	*P*	Corr.	*P*	Corr.	*P*	Corr.	*P*
BRIC	−0.12	<0.001	−0.25	<0.001	−0.17	<0.001	−0.27	0.02	−0.29	<0.05
Community Capital	−0.08	<0.01	−0.13	<0.001	0.01	0.929	0.05	0.71	−0.16	0.26
Economic	0.16	<0.001	−0.09	<0.05	0.15	<0.001	−0.10	0.42	−0.09	0.54
Environment	−0.11	<0.001	−0.10	<0.01	−0.09	<0.001	0.09	0.45	0.12	0.41
Infrastructure	−0.18	<0.001	−0.02	0.54	−0.35	<0.001	−0.41	<0.01	−0.33	<0.05
Institution	0.16	<0.001	−0.11	<0.001	0.18	<0.001	0.03	0.79	−0.20	0.15
Social	−0.24	<0.001	−0.30	<0.001	−0.35	<0.001	−0.51	<0.001	−0.42	<0.001

In urban areas (*n* = 1,101), mean stroke percentage was 5.87% (SD = 1.24%). Negative correlations were found for social (*r* = −0.30, *p* < 0.001) and environmental resilience (*r* = −0.096, *p* = < 0.01). In transitioning areas (rural to urban, *n* = 72), mean stroke burden (% of Medicare FFS beneficiaries) was 5.69% (SD = 1.46%). Social (*r* = −0.505, *p* < 0.001) and infrastructural resilience (*r* = −0.408, *p* < 0.001) were inversely correlated with stroke. In deurbanizing areas (urban to rural, n = 52), mean stroke percentage was 5.79% (SD = 1.40%). Social (*r* = −0.424, *p* = < 0.01) and infrastructural resilience (*r* = −0.327, *p* < 0.05) were negatively correlated. Please refer to Tables 2, 3 for a more detailed breakdown of the results.

Table 4 shows the correlation results of the resilience metrics change in stroke burden (percentage point difference, 2022–2014). Across all counties the mean increase in stroke burden (percentage point difference, 2022–2014) was 2.17% (SD = 1.90%). Negative correlations were observed with change in stroke burden (percentage point difference, 2022-2014) and BRIC (*r* = −0.13, *p* < 0.001), community capital (*r* = −0.07, *p* < 0.001), environment (*r* = −0.07, *p* < 0.001), infrastructure (*r* = −0.13, *p* < 0.001), and social capital (*r* = −0.14, *p* < 0.001). In rural counties, the mean increase in stroke burden (percentage point difference, 2022–2014) was 2.13% (SD = 1.32%), with inverse correlations for change in stroke burden (percentage point difference, 2022–2014) and BRIC (*r* = −0.13, *p* < 0.001), environment (*r* = −0.09, *p* < 0.001), infrastructure (*r* = −0.18, *p* < 0.001), institution (*r* = 0.05, *p* < 0.05), and social capital (*r* = −0.16, *p* < 0.001). In urban counties, the mean increase in stroke burden (percentage point difference, 2022–2014) was 2.24% (SD = 0.96%). Negative correlations were observed for change in stroke burden (percentage point difference, 2022–2014) and BRIC (*r* = −0.21, *p* < 0.001), community capital (*r* = −0.10, *p* < 0.01), economic capital (*r* = −0.16, *p* < 0.001), infrastructure (*r* = −0.06, *p* < 0.05), institution (*r* = −0.09, *p* < 0.01), and social capital (*r* = −0.19, *p* < 0.001). In transitioning areas (rural to urban), the mean increase in stroke burden (percentage point difference, 2022–2014) was 2.25% (SD = 1.02%), with social capital (*r* = −0.26, *p* < 0.05) inversely correlated with this change. In deurbanizing areas (urban to rural, n = 52), the mean increase in stroke burden (percentage point difference, 2022–2014) was 2.06 (SD = 1.14), with BRIC (*r* = −0.25, *p* = 0.075), institution (*r* = −0.32, *p* < 0.01), and social capital (*r* = −0.37, *p* < 0.01) showing negative correlations with this change.

**Table 4:** Correlation results of study variables with change in stroke burden (percentage point difference, 2022–2014).

	Overall (*n* = 3,100)		Urban (*n* = 1,101)		Rural (*n* = 1,875)		Transitioning (rural to urban) (*n* = 72)		Urban to rural (deurbanizing) (*n* = 52)	
	Corr.	*P*	Corr.	*P*	Corr.	*P*	Corr.	*P*	Corr.	*P*
BRIC	−0.13	*P* < 0.001	−0.21	*P* < 0.001	−0.13	<0.001	−0.15	0.03	−0.25	0.08
Community Capital	−0.07	*P* < 0.001	−0.10	*P* < 0.01	−0.05	<0.01	0.07	0.561	−0.14	0.33
Economic	0.01	0.64	−0.16	*P* < 0.001	0.04	<0.001	−0.11	0.357	−0.12	0.38
Environment	−0.07	*P* < 0.001	−0.05	0.078	−0.09	<0.001	0.08	0.493	0.14	0.33
Infrastructure	−0.13	*P* < 0.001	−0.06	*P* < 0.001	−0.18	<0.001	−0.21	0.074	−0.20	0.16
Institution	0.02	0.22	−0.09	*P* < 0.01	0.05	<0.06	−0.14	0.257	−0.32	*P* < 0.01
Social	−0.14	*P* < 0.001	−0.19	*p* < 0.001	−0.16	<0.001	−0.26	*P* < 0.05	−0.37	*P* < 0.01

Table 5 presents the results of multivariable regression models predicting stroke burden (% of Medicare FFS beneficiaries) in 2022 and change in stroke burden (percentage point difference, 2022–2014). Social resilience emerged as the strongest negative predictor across models. Economic and institutional resilience were consistently positive predictors. Environmental and infrastructural resilience also showed negative associations, though their significance varied by different county groups. Notably, in deurbanizing and transitioning counties, only social resilience remained statistically significant.

**Table 5:** Linear regression predicting stroke burden (% of Medicare FFS beneficiaries) in 2022 and change in stroke burden (percentage point difference, 2022–2014).

Characteristics	All counties	Rural	Urban	Transitioning (rural to urban)	Deurbanizing (urban to rural)
	*N* = 3,100	*N* = 1875	*N* = 1,101	*N* = 72	*N* = 52
Outcome 1: stroke burden 2022 (% of Medicare FFS beneficiaries)					
Social	*β* = −10.484*, 95% CI: (−11.853, −9.115) SE = 0.698	*β* = −9.114*, (−10.972, −7.257) SE = 0.948	*β* = −9.672*, 95% CI: (−11.624, −7.720) SE = 0.996	*β* = −11.429*, 95% CI: (−18.866, −3.991), SE = 3.795	*β* = −13.438*, 95% CI: (−26.226, −0.650) SE = 6.524
Economic	*β* = 9.536*, 95% CI: (8.202, 10.871) SE = 0.681	*β* = 7.238*, 95% CI: (5.570, 8.907) SE = 0.851	*β* = 3.339*, 95% CI: (0.632, 6.045) SE = 1.381	–	–
Infrastructure	*β* = −2.825*, 95% CI: (−4.062, −1.588), SE = 0.631	*β* = −7.180*, 95% CI: (−8.907, −5.454), SE = 0.881	–	*β* = −4.143, 95% CI: (−12.238, 3.953), SE = 4.130	*β* = −1.624, 95% CI: (−12.145, 8.897), SE = 5.368
Community Capital	*β* = −0.984, 95% CI: (−2.016, 0.047), SE = 0.526	–	*β* = −2.225*, 95% CI: (−3.780, −0.669), SE = 0.794	–	–
Institution	*β* = 5.754*, 95% CI: (4.032, 7.476), SE = 0.879	*β* = 6.313*, 95% CI: (4.191, 8.434), SE = 1.082	*β* = −1.836, 95% CI: (−5.017, 1.344), SE = 1.623	–	–
Environment	*β* = −2.381*, 95% CI: (−3.568, −1.193), SE = 0.606	*β* = −2.048*, 95% CI: (−3.818, −0.277), SE = 0.903	*β* = −1.768*, 95% CI: (−3.247, −0.290), SE = 0.754	–	–
Outcome 2: Change in stroke burden (percentage point difference, 2022–2014)					
Social	*β* = −2.09*, 95% CI: (−3.204, −0.969), SE = 0.57	*β* = −0.85, 95% CI: (−4.019, 1.072), SE = 0.569	*β* = −0.85, 95% CI: (−2.46, 0.75), SE = 0.63	*β* = −4.904*, 95% CI: (−9.325, −0.485), SE = 2.216	*β* = −8.823*, 95% CI: (−16.452, −1.194), SE = 3.796
Economic	*β* = 0.57, 95% CI: (−0.35, 1.5), SE = 0.45	–		–	–
Infrastructure	*β* = −2.33*, 95% CI: (−3.418, −1.242), SE = 0.555	*β* = −2.9*, 95% CI: (−5.271, −0.854), SE = 0.832	*β* = −1.18, 95% CI: (−3.45, 1.08), SE = 0.66	–	–
Community Capital	*β* = −1.07*, 95% CI: (−1.967, −0.168), SE = 0.459	*β* = −0.04, 95% CI: (−1.394, 1.323), SE = 0.692	*β* = −1.08, 95% CI: (−2.31, 0.15), SE = 0.55	–	–
Institution	–	*β* = 0.73, 95% CI: (−0.618, 2.078), SE = 0.83	*β* = −0.42, 95% CI: (−2.15, 1.19), SE = 0.95	–	*β* = −10.92, 95% CI: (−23.21, 1.38), SE = 6.12
Environment	*β* = −2.54*, 95% CI: (−3.562, −1.511), SE = 0.523	*β* = −3.03*, 95% CI: (−5.204, −1.859), SE = 0.853	*β* = 1.03, 95% CI: (−1.13, 3.18), SE = 1.2	–	–

*means statistical significance <0.05.

We evaluated regression assumptions across all models. Global F-tests indicated that the predictors jointly improved model fit across all county types. Residual fitted and Q-Q plot inspections showed no major violations of homoscedasticity or normality, and deviance values were proportional to sample size. Influence diagnostics did not identify any observations with undue leverage. For the Stroke Burden 2022 (% of Medicare FFS beneficiaries) prediction, model performance varied across county types, with *R*^2^-values ranging from 0.115 (urban-to-rural transitioning counties) to 0.266 (rural-to-urban transitioning counties). The overall model explained approximately 17.5% of the variation in county-level stroke burden (adjusted R^2^ = 0.173). Variance inflation factors (VIFs) were used to assess multicollinearity among BRIC domains. Across all models, VIF values remained low (between 1.07 and 1.98). This indicates that collinearity did not meaningfully inflate standard errors. Even in small subgroups (e.g., transitioning counties), VIFs remained below conventional thresholds (VIF < 2).

For the regression models predicting change in stroke burden (percentage point difference, 2022–2014) adjusted R^2^ values ranged from 0.032 (overall) to 0.159 (deurbanizing counties). Rural, urban, and transitioning models exhibited adjusted R^2^ values between 0.045 and 0.052, indicating that BRIC domains explained a small but meaningful proportion of variation in stroke burden change. VIFs were low (<2) across all models. Detailed bivariate regression results for each resilience domain, including *β* estimates, standard errors, and 95% confidence intervals across all county strata, are presented in Supplementary File 2.

**Figure 2:**
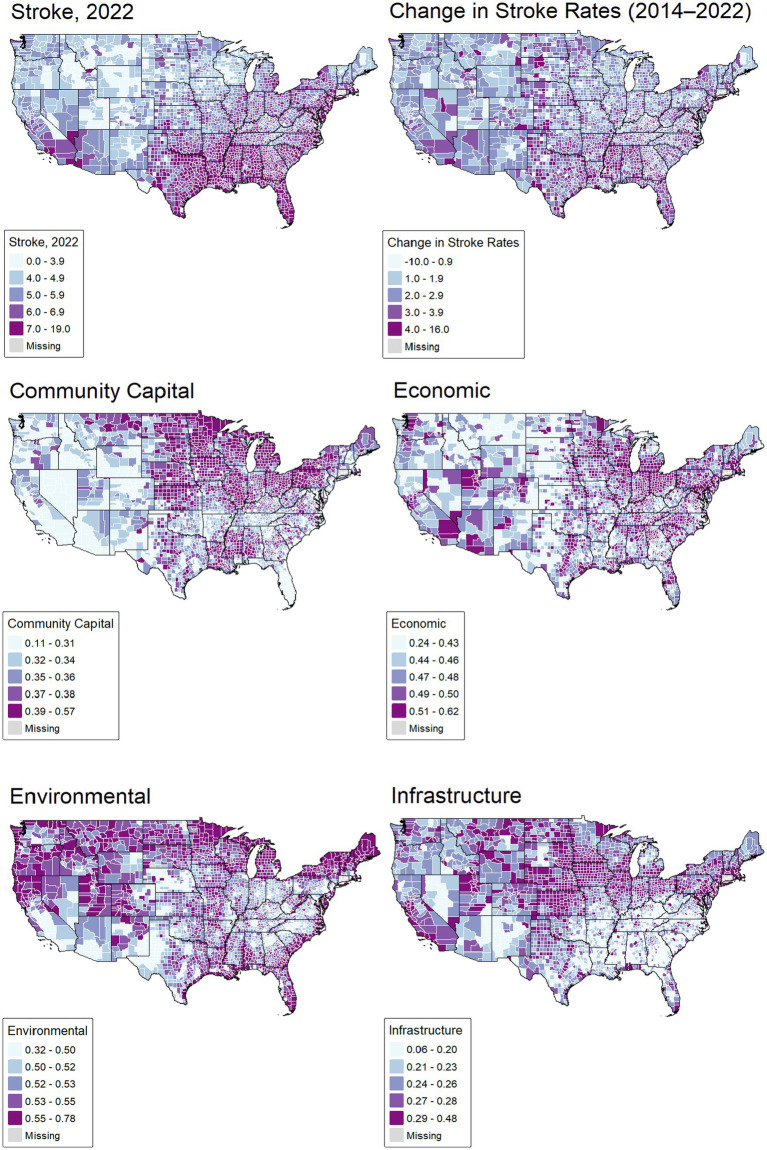
The spatial distribution of study variables.

Figure 2 shows that stroke burden (% of Medicare FFS beneficiaries) and the burden change are unevenly distributed across the U. S., with the highest rates concentrated in the Southeast. The map highlights how geographic disparities in stroke mirror broader socioeconomic and infrastructural inequalities.

**Figure 3:**
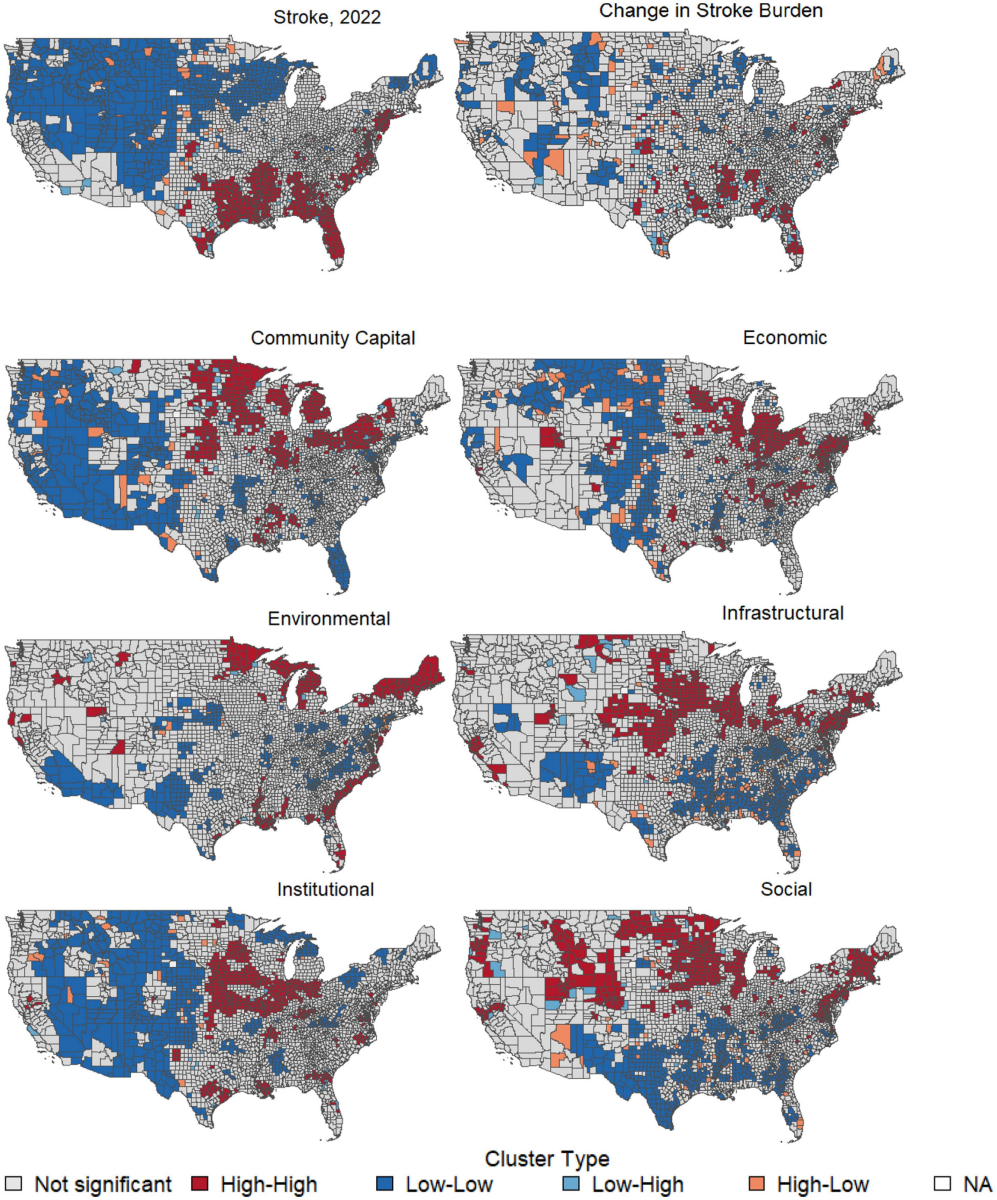
LISA maps. LISA maps are based on a first-order queen contiguity weight matrix. High-high clusters represent counties with high values surrounded by high-value neighbors; low-low clusters reflect the opposite.

Figure 3 highlights spatial clustering of stroke burden (% of Medicare FFS beneficiaries). In the LISA framework, high-high clusters represent counties with high stroke burden that are surrounded by neighboring counties that also exhibit high values, indicating localized hot spots. Low-low clusters reflect counties with low stroke burden that are adjacent to neighbors with similarly low values, which form cold spots. High-low clusters indicate counties with high values surrounded by low-value neighbors, while low-high clusters represent counties with low values surrounded by high-value neighbors; these two categories identify spatial outliers. High-high clusters (high stroke burden and high increase in change in stroke burden from 2014 to 2022) appear in the Southeast, where counties with elevated stroke burden (% of Medicare FFS beneficiaries) are surrounded by similarly high-burden neighbors. Low-low clusters, in contrast, are found in parts of the Midwest and Northeast, where stroke burden (% of Medicare FFS beneficiaries) remains relatively low. This highlights that stroke burden (% of Medicare FFS beneficiaries) is geographically concentrated rather than randomly distributed.

Global Moran’s I statistics indicated significant positive spatial autocorrelation for both the stroke outcomes and the resilience indicators, confirming clear geographic clustering across U. S. counties. Stroke burden in 2022 showed strong clustering (Moran’s I = 0.603, *p* < 0.001), indicating that high-burden counties tend to be adjacent to other high-burden counties, while low-burden counties similarly cluster together. Change in stroke burden from 2014 to 2022 exhibited moderate clustering (Moran’s I = 0.252, *p* < 0.001), suggesting that increases in stroke burden follow regional spatial patterns rather than occurring randomly.

The BRIC overall resilience index demonstrated even stronger spatial clustering (Moran’s I = 0.653, *p* < 0.001), reflecting pronounced geographic structuring in community resilience capacity. Among the individual domains, social resilience showed substantial clustering (Moran’s I = 0.561, *p* < 0.001), indicating that social determinants of resilience tend to concentrate regionally. The economic domain also exhibited significant clustering (Moran’s I = 0.508, *p* < 0.001). Infrastructure resilience followed a similar spatial pattern with strong clustering (Moran’s I = 0.558, *p* < 0.001), and community capital showed comparably high clustering (Moran’s I = 0.574, *p* < 0.001), highlighting the regional concentration of civic and community resources. The institutional resilience domain demonstrated one of the strongest clustering patterns (Moran’s I = 0.642, *p* < 0.001), reflecting the geographic structuring of governance and administrative capacity. The environmental resilience domain also exhibited significant spatial clustering (Moran’s I = 0.551, *p* < 0.001).

## Discussion

This study examined the role of community resilience in shaping geographic disparities in stroke burden (% of Medicare FFS beneficiaries) among Medicare beneficiaries across rural, urban, transitioning, and deurbanizing U. S. counties. Overall, we observed that several resilience metrics including social and infrastructural, tended to show protective associations with stroke, while economic and institutional domains were sometimes positively related. However, these associations were not consistent across all county types. These findings highlight that the role of resilience varies depending on local context. Also, spatial analysis showed important geographic patterns, with high stroke county clusters concentrated in the southeastern “Stroke Belt,” which align with counties of low resilience across multiple domains. It is important to note, however, that the transitioning (*n* = 72) and deurbanizing (*n* = 52) county groups were relatively small compared with the stable rural and urban categories. Therefore, results for these groups should be interpreted with caution. These findings suggest that community resilience is not a uniform construct but operates differently across its dimensions, with certain domains serving as protective factors while others may reflect structural or contextual vulnerabilities in some areas. Study findings show the need to consider resilience as a multidimensional determinant of health that can be used to explain why some counties experience disproportionately higher stroke burden (% of Medicare FFS beneficiaries).

More specifically, social resilience emerged as the most consistent protective factor in this study and was negatively associated with stroke burden (% of Medicare FFS beneficiaries) and changing stroke burden (percentage-point difference, 2022–2014). The BRIC social domain includes determinants directly tied to health, including educational attainment, transportation and communication capacity, health insurance coverage, food security, physician availability, and mental health supports (19). These elements map closely onto known risk pathways for stroke, particularly for older adults who depend heavily on reliable access to care and social support. This geographic divergence suggests that social resilience is not only statistically protective but also spatially patterned in ways that mirror the nation’s most persistent stroke disparities. These indicators go beyond interpersonal ties to reflect structural conditions that directly influence access to care, prevention, and recovery resources.

Spatial analysis reinforced this pattern. Deficits in social resilience clustered in the Southeastern Stroke Belt and showed low-low clusters of counties in the Southeastern Stroke Belt. This overlapped with high-high stroke burden (% of Medicare FFS beneficiaries) counties. High-high clusters of social resilience were observed in the Upper Midwest, Mountain West, and Northeast, where stroke burden (% of Medicare FFS beneficiaries) was lower. These geographic patterns suggest that the persistence of stroke disparities in the Southeast is partly linked to deficits in fundamental social determinants of health, such as education, transportation, and health care access.

Our findings align with research that documented multiple BRIC social elements map onto stroke mechanisms. For example, higher educational attainment is associated with lower incident stroke and better outcomes (24, 25). Similarly, health insurance coverage is associated with improved in-hospital survival and care processes in stroke populations. This reinforces why counties with stronger coverage tend to perform better (26). Access enabling factors including transportation and communication capacity, also have direct clinical relevance. For example, longer prehospital transport times worsen functional outcomes while widespread telehealth infrastructure improve care in resource-limited regions (27, 28).

Limited English proficiency is linked to poorer preventive stroke care and anticoagulation management. Because limited English proficiency is one of the indicators included in the BRIC social resilience domain, its relevance to stroke outcomes is important to clarify. Limited English proficiency is linked to poorer preventive stroke care, reduced access to guideline-recommended treatments, and delays in receiving time-sensitive stroke interventions (29). This explains why counties with stronger English proficiency show advantages (30). On the population-health side, mental health support matters because depression elevates stroke incidence and mortality. Communities with better psychosocial resources may mitigate that risk (31, 32). Finally, physician access is broadly associated with lower population mortality (33, 34). Overall, the literature indicates that BRIC’s social domain bundles health proximal determinants that plausibly drive the protective spatial pattern that we found.

Infrastructure resilience was also negatively associated with stroke burden (% of Medicare FFS beneficiaries). The BRIC infrastructure domain includes hospital and bed availability, shelter capacity, broadband access, and transportation networks, critical for enabling stroke care and follow-up. Geographic disparities in infrastructure often mirror patterns of health access. Disadvantaged communities experience lower infrastructure access, which can impede mobility and care delivery (35). Spatial clustering showed low-low clusters of infrastructure resilience in the Southeast, overlapping with stroke high-high clusters, while high-high resilience clusters were concentrated in the Mid-Atlantic, Upper Midwest, and portions of the Northeast. These results highlight the importance of physical and digital infrastructure in resilience.

Both Environmental and community capital domains were inversely associated with stroke burden (% of Medicare FFS beneficiaries), though less consistently than social or infrastructural domains. Community capital (e.g., civic engagement, place attachment) fosters social cohesion and collective health, while environmental resilience (e.g., local food systems, natural buffers) supports stability and wellbeing. Low-low clusters for both domains overlapped with the Stroke Belt. This aligned with counties of elevated stroke burden (% of Medicare FFS beneficiaries), while high-high clusters of resilience were evident in the West, Northern Great Plains, and Northeast. These results emphasize how ecological stability, community engagement, and collective community resources contribute to health equity. Prior studies have shown that community networks and capital are particularly important for resilience in older adults. McKibbin et al. (12) and Wells (13, 14) found that community engagement and health status were strong predictors of resilience. Meanwhile, (36) reported that environmental and community measures in BRIC can vary substantially across counties. Our study builds on this by showing that where environmental and community capital resilience are strong, stroke burden (% of Medicare FFS beneficiaries) is consistently lower.

In contrast, economic and institutional resilience were positively associated with stroke burden (% of Medicare FFS beneficiaries), an unexpected finding. Spatial clustering showed that high-high clusters of both domains were concentrated in urbanized regions, including the Northeast Corridor, Midwest metropolitan centers, and coastal areas, many of which coincided with high-high stroke clusters in the Stroke Belt. Because BRIC was designed as a natural hazard preparedness metric rather than a health service focused indicator, the positive associations observed for economic and institutional resilience are more appropriately interpreted as measurement or contextual artifacts rather than reflecting causal mechanisms. These domains may capture structural capacity, governance, or economic activity that co-occur with older population composition, higher comorbidity burdens, or better diagnostic and reporting infrastructure. Evans et al. (3) reported persistent clustering of stroke mortality in the Southeast, and Koton et al. (39) found that national declines in stroke incidence have not erased geographic disparities. Camacho et al. (36) similarly warned that BRIC’s economic and institutional measures sometimes may produce paradoxical associations. At a national level, both domains correlated positively with stroke burden (% of Medicare FFS beneficiaries), yet within urban counties, these associations were weaker or reversed. Economic resilience (e.g., employment, business structure) and institutional resilience (e.g., mitigation spending, governance capacity) are not health service indicators. Instead, they may co-occur with older, comorbid urban populations and reactive policy investments. This suggests that these domains serve as structural markers rather than direct protective factors for stroke. Similar cautions about applying BRIC’s economic and institutional domains to chronic disease contexts have been raised in the literature (36, 37).

Some resilience domains showed little or inconsistent links with stroke across the rural-urban continuum and did not form clear spatial patterns outside of the Stroke Belt. This suggests that resilience works differently depending on local context and may not affect all domains in the same way. Earlier studies also point to this variability. McKibbin et al. (12) and Wells (13, 14) found that resilience was tied more strongly to mental and physical health than to structural factors. Camacho et al. (36) noted problems with how BRIC is applied across communities, and Anderson’s RISE model highlighted that resilience is multidimensional and context-specific (38). The lack of consistent findings here shows the importance of using local, tailored approaches when studying and strengthening resilience.

Despite the important findings, our study has several limitations. Stroke burden was derived from Medicare claims, which may introduce misclassification because diagnoses depend on billing practices rather than clinical confirmation. It remains ecological, so individual-level inferences are not possible. BRIC’s design for hazard resilience means economic and institutional domains may not align with healthcare needs. Therefore, the social resilience domain that is composed of multiple health-proximal indicators may partly explain its stronger associations. We defined county “trajectory” by comparing RUCC codes from 2010 and 2020. Because relatively few counties shifted from being rural to urban or being urban to rural, estimates for these small groups might be less accurate due to smaller sample size. In addition, some exclusions and recoding (e.g., removing Connecticut planning regions) might affect the generalizability of the findings to all U. S. mainland counties. Additionally, the transitioning and deurbanizing groups comprised fewer than 3% of U. S. counties combined, which may have reduced statistical power and widened confidence intervals for those subgroup analyses. As such, these results should be viewed as exploratory and hypothesis generating rather than conclusive.

However, because BRIC aggregates these demographic and socioeconomic characteristics into composite domains, the independent effects of age, race/ethnicity, and sex cannot be disentangled from the resilience measures; this represents an inherent limitation of using BRIC in epidemiologic models. Our regression models did not explicitly account for spatial dependence. Stroke burden and community resilience indicators cluster geographically, and unmeasured spatial autocorrelation could bias standard errors or attenuate true associations. In addition, BRIC domains may mask heterogeneity within counties, where neighborhood-level conditions could differ substantially from county averages. Finally, because the stroke outcome is derived from Medicare fee-for-service beneficiaries, findings may not generalize to younger populations or those enrolled in Medicare Advantage or other insurance types.

## Conclusion

The BRIC social domain was the most consistent protective factor for both current burden and change over time in stroke burden (% of Medicare FFS beneficiaries) among Medicare beneficiaries; infrastructure was also protective, particularly outside large metropolitan areas. By contrast, economic and institutional domains related positively to stroke burden (% of Medicare FFS beneficiaries) in the overall sample and rural models. Findings highlight that indicators designed for natural hazard may reflect structural context or detection rather than health-proximal access in older populations. Spatially, high-high stroke clusters in the Southeast were associated with low social and infrastructural resilience. Findings highlight the importance of moving beyond static rural/urban county classifications to trajectory-oriented planning that prioritizes the most health-proximal measures including coverage and care navigation, transportation, language/communication, food and housing stability, broadband, and health care provider capacity. This might help mitigate the persistent geographic disparities in stroke among older adults. Policy might need to consider following county trajectory and the LISA hot/cold spots rather than one size fits all rural/urban interventions.

## Data Availability

Publicly available datasets were analyzed in this study. This data can be found at: https://www.policymap.com/.
