# Supplementary material for: Community resilience and stroke outcomes in older adults: beyond rural-urban classifications
Source: Front Public Health. 2025 Dec 12;13:1700006. doi: 10.3389/fpubh.2025.1700006 (PMC12742400; doi:10.3389/fpubh.2025.1700006)
Supplement: Supplementary file 2 [file Supplementary_file_2.docx]

**Supplementary Material**

Bivariate linear regression models were used to examine each BRIC domain separately as a predictor of each outcome.

**Supplementary Table 1.** Bivariate Linear Regression Predicting Stroke Burden (2022) and Change in Stroke Burden (2014-2022) Across U.S. Counties, Overall and by Rural-Urban Classification, N=3,100

| **Characteristics** | **All counties** | **Rural** | **Urban** | **Transitioning**  **(Rural to urban)** | **Deurbanizing**  **(Urban to rural)** |
| --- | --- | --- | --- | --- | --- |
|  | **N=3,100** | **N=1,875** | **N=1,101** | **N=72** | **N=52** |
| **Outcome 1: Stroke Burden 2022 (% of Medicare FFS beneficiaries)** |  |  |  |  |  |
| Social | β = -7.496*,  95% CI: (-8.554, -6.439)  SE = 0.540 | β = -11.912*,  95% CI: (-13.374, -10.450)  SE = 0.746 | β = -8.610*,  95% CI: (-10.228, -6.992)  SE = 0.826 | β = -13.897*,  95% CI: (-19.559, -8.236)  SE = 2.839 | β = -14.828*,  95% CI: (-23.822, -5.833)  SE = 4.478 |
| Economic | β = 5.500*,  95% CI: (4.335, 6.665)  SE = 0.594 | β = 5.683*,  95% CI: (4.011, 7.355)  SE = 0.853 | β = -3.629*,  95% CI: (-5.955, -1.303)  SE = 1.187 | β = -4.368,  95% CI: (-15.062, 6.326)  SE = 5.362 | β = -2.763,  95% CI: (-11.810, 6.285)  SE = 4.504 |
| Infrastructure | β = -5.143*,  95% CI: (-6.159, -4.127)  SE = 0.519 | β = -11.485*,  95% CI: (-12.887, -10.083)  SE = 0.715 | β = -0.458,  95% CI: (-1.926, 1.010)  SE = 0.749 | β = -12.211*,  95% CI: (-18.729, -5.692)  SE = 3.269 | β = -9.407*,  95% CI: (-17.129, -1.685)  SE = 3.845 |
| Community Capital | β=-0.984, 95% CI: (-2.016, 0.047), SE=0.526 | β = 0.070,  95% CI: (-1.468, 1.608)  SE = 0.785 | β = -3.515*,  95% CI: (-5.079, -1.951)  SE = 0.798 | β = 1.251,  95% CI: (-5.352, 7.853)  SE = 3.310 | β = -5.108,  95% CI: (-14.017, 3.801)  SE = 4.436 |
| Institution | β = 7.612*,  95% CI: (5.931, 9.293)  SE = 0.858 | β = 8.672*,  95% CI: (6.476, 10.868)  SE = 1.121 | β = -6.145*,  95% CI: (-9.360, -2.929)  SE = 1.642 | β = 1.620,  95% CI: (-10.516, 13.755)  SE = 6.085 | β = -11.383,  95% CI: (-27.063, 4.296)  SE = 7.806 |
| Environment | β = -3.800*,  95% CI: (-4.995, -2.605)  SE = 0.610 | β = -3.656*,  95% CI: (-5.573, -1.739)  SE = 0.978 | β = -2.414*,  95% CI: (-3.890, -0.938)  SE = 0.753 | β = 2.549,  95% CI: (-4.184, 9.283)  SE = 3.376 | β = 3.686,  95% CI: (-5.072, 12.444)  SE = 4.360 |
| **Outcome 2: Change in stroke burden (percentage point difference, 2022-2014)** |  |  |  |  |  |
| Social | β = -3.601*,  95% CI: (-4.476, -2.725)  SE = 0.448 | β = -4.596*,  95% CI: (-5.907, -3.285)  SE = 0.669 | β = -4.141*, 95% CI: (-5.435, -2.847), SE = 0.660 | β = -10.628*, 95% CI: (-17.955, -3.301), SE = 3.738 | β = -10.628*, 95% CI: (-17.955, -3.301), SE = 3.738 |
| Economic | β = 0.228,  95% CI: (-0.732, 1.188)  SE = 0.490 | β = 1.198,  95% CI: (-0.243, 2.639)  SE = 0.735 | β = -4.947*, 95% CI: (-6.735, -3.159), SE = 0.912 | β = -3.243, 95% CI: (-10.402, 3.916), SE = 3.655 | β = -3.243, 95% CI: (-10.402, 3.916), SE = 3.655 |
| Infrastructure | β = -3.011*,  95% CI: (-3.844, -2.178)  SE = 0.425 | β = -5.080*,  95% CI: (-6.333, -3.827)  SE = 0.639 | β = -1.237*, 95% CI: (-2.372, -0.100), SE = 0.580 | β = -4.668, 95% CI: (-11.035, 1.699), SE = 3.248 | β = -4.668, 95% CI: (-11.035, 1.699), SE = 3.248 |
| Community Capital | β = -1.757*,  95% CI: (-2.645, -0.869)  SE = 0.453 | β = -1.442*,  95% CI: (-2.752, -0.132)  SE = 0.668 | β = -1.979*, 95% CI: (-3.197, -0.761), SE = 0.621 | β = -3.549, 95% CI: (-10.657, 3.559), SE = 3.627 | β = -3.549, 95% CI: (-10.657, 3.559), SE = 3.627 |
| Institution | β = 0.873,  95% CI: (-0.512, 2.258)  SE = 0.707 | β = 2.017*,  95% CI: (0.117, 3.917)  SE = 0.969 | β = -3.743*, 95% CI: (-6.245, -1.240), SE = 1.276 | β = -14.709*, 95% CI: (-26.759, -2.659), SE = 6.152 | β = -14.709*, 95% CI: (-26.759, -2.659), SE = 6.152 |
| Environment | β = -2.030*,  95% CI: (-3.006, -1.054)  SE = 0.498 | β = -3.293*,  95% CI: (-4.927, -1.659)  SE = 0.833 | β = -1.033, 95% CI: (-2.182, 0.116), SE = 0.586 | β = 3.505, 95% CI: (-3.440, 10.450), SE = 3.543 | β = 3.505, 95% CI: (-3.440, 10.450), SE = 3.543 |

**Note:** Domains identified as statistically significant in the bivariate models were carried forward into multivariable modeling.
